# In-Depth Analysis of the Peripheral Immune Profile of HER2+ Breast Cancer Patients on Neoadjuvant Treatment with Chemotherapy Plus Trastuzumab Plus Pertuzumab

**DOI:** 10.3390/ijms25179268

**Published:** 2024-08-27

**Authors:** Ayelén Ivana Pesce Viglietti, María Belén Bordignon, Alexis Ostinelli, Manglio Miguel Rizzo, Gerardo Cueto, María Belén Sanchez, Florencia Perazzo, Mora Amat, Federico Coló, María Victoria Costanzo, Adrián Nervo, Jorge Nadal, Gabriel Crimi, Ignacio Mc Lean, Eunice Amancay Spengler, José Mordoh, Pablo Mandó, Estrella Mariel Levy

**Affiliations:** 1 Centro de Investigaciones Oncológicas (FUCA), Fundación Cáncer, Ciudad Autónoma de Buenos Aires C1426AOE, Argentina; ayelenpv@gmail.com (A.I.P.V.); bordignonmbelen@gmail.com (M.B.B.);; 2 Instituto Alexander Fleming, Ciudad Autónoma de Buenos Aires C1426AOE, Argentina; 3 Clinical Oncology Unit, Hospital Universitario Austral, Derqui-Pilar, Buenos Aires B1629ODT, Argentina; 4 Grupo de Bioestadística Aplicada, Departamento de Ecología, Genética y Evolución, Instituto de Ecología, Genética y Evolución de Buenos Aires (IEGEBA-UBA/CONICET), Facultad de Ciencias Exactas y Naturales, Universidad de Buenos Aires, Ciudad Autónoma de Buenos Aires C1428AOE, Argentina; 5 Centro de Educación Médica e Investigaciones Clínicas (CEMIC), Ciudad Autónoma de Buenos Aires C1431AOE, Argentina

**Keywords:** HER2+ breast cancer, neoadjuvant treatment, trastuzumab, pertuzumab, immune profile

## Abstract

Currently, therapy for early-stage human epidermal growth factor receptor 2-positive (HER2+) breast cancer (BC) is based on the combination of trastuzumab and pertuzumab plus chemotherapy in a neoadjuvant regimen. The INMUNOHER study aimed to detect immunological markers in peripheral blood and their association with treatment response. Sixty-two HER2+ BC patients were recruited. Pre-treatment samples were obtained before the start of treatment, while post-treatment samples were obtained after completing therapy and before surgery and were analyzed by flow cytometry. The pathologic complete response (pCR) rate achieved was 82.3%. The expression of the NKp30, PD-1, and TIM-3 receptors was reduced in the Natural Killer (NK)-CD56dim subset of patients who did not achieve pCR. Following therapy, many changes were found in leukocytes, including alterations in T cell lymphocyte proportions. Also, the percentage of NK cells decreased, and several phenotypic changes were observed in this population. After treatment, IFN-γ production by NK cells against HER2+-cells with or without trastuzumab was significantly reduced. HER2-targeted therapy plus chemotherapy demonstrated high efficacy in most patients, reducing the statistical power for finding immunological markers. However, NK subset phenotypes correlated better with response groups, and numerous changes in the percentage of leukocytes and T and NK cells, as well as changes in the functionality of NK cells, were observed in most patients after treatment, encouraging further research into these immune populations.

## 1. Introduction

Human epidermal growth factor receptor 2-positive (HER2+) breast cancer (BC), constituting around 20% of all BC, is characterized by the amplification of the *ERBB2* gene on chromosome 17q12, resulting in an overexpression of the HER2 protein [1]. This overexpression activates cell growth and proliferation pathways, such as the PI3K and MAPK pathways [2]. Prior to the development of HER2-targeted therapies (HER2TTs), HER2+ BCs were known for their highly proliferative and aggressive nature, leading to a poor prognosis [3].

The first approved HER2TT, trastuzumab, targets a specific region in subdomain IV of the extracellular portion of the HER2 protein. Its mechanism involves the suppression of HER2 intracellular signaling, and its human IgG1 isotype framework is purposely designed to stimulate antibody-dependent cell-mediated cytotoxicity (ADCC) [4]. This process relies on fragment crystallizable (Fc) receptors (e.g., *FCGR3A*/CD16) expressing effector cells that recognize either autologous or therapeutic antibodies bound to a target cell, thereby inducing cell death through the perforin–granzyme system [5]. Immune cells such as Natural Killer (NK) cells, gamma-delta T cells, and monocytes are capable of engaging IgG antibodies like trastuzumab in this process [6,7]. In vitro research has indicated a correlation between trastuzumab-mediated ADCC and patient immune responses to trastuzumab treatment [8,9,10].

Considering that HER2 signaling activation relies on receptor homodimerization or heterodimerization with other members of the HER family of receptors, a second anti-HER2 antibody was developed. Pertuzumab is a monoclonal antibody (mAb) that inhibits the formation of the relevant HER2/HER3 heterodimer by binding to the dimerization domain of the HER2 receptor [11,12,13]. This antibody has shown effectiveness in activating ADCC comparable to trastuzumab [14]. When pertuzumab and trastuzumab are combined to treat HER2+ primary tumors, response rates increase significantly [15,16].

Extensive translational studies have been conducted to identify potential biomarkers of pathologic complete response (pCR). These biomarkers could be utilized to identify specific patient groups for de-escalation studies or, conversely, to develop new treatments for patient subgroups inherently resistant to therapy. Tumor molecular profiling has revealed certain indicators of higher rates of pCR, such as lower estrogen receptor expression, elevated levels of HER2 amplification, and the HER2-enriched PAM50 molecular subtype [17,18,19]. Additionally, several immune-related biomarkers, including tumor-infiltrating lymphocyte levels and immune gene signatures, have been reported to identify patients with increased rates of pCR [18,20,21,22,23,24,25]. Furthermore, clinical markers such as hormone receptor (HR) expression and Ki67 values have been associated with a greater or lesser likelihood of response [26,27,28]. However, it is still unclear whether immune populations assessed in the periphery can contribute to the identification of patients who will or will not respond to HER2TT. The literature reveals that 60.8% of patients with HER2+ BC attain a pCR [29]. This implies that relying only on intratumoral HER2 expression does not enable clinicians to accurately determine which patients will benefit from trastuzumab/pertuzumab. This challenge underscores the need for our study.

INMUNOHER was a prospective trial designed to assess circulating/peripheral immune markers in neoadjuvant therapy against HER2 in BC patients. We hypothesized that the configuration of the circulating immune repertoire could be associated with several clinical variables and the efficacy of HER2TT. In this study, we sought to determine whether peripheral blood (PB) immune profiles at baseline or their changes over time could predict responses to HER2TT. Additionally, we performed a multiparametric immune analysis on NK and T cells to deeply characterize groups associated with clinical/histologic markers and BC subgroups inside the HER2+ subtype.

## 2. Results

### 2.1. Patient Characteristics and Clinical Responses

Sixty-two HER2+ BC women patients from the Alexander Fleming Institute (*n* = 43), CEMIC University Hospital (*n* = 14), and Austral University Hospital (*n* = 5) were recruited between April 2018 and October 2021. Clinical characteristics are described in Table 1. Patients were classified into two groups according to their best clinical response: the responder group comprised 51 patients (82.3%) who achieved pCR, while the non-responder group comprised 11 patients (17.7%) who did not achieve pCR after neoadjuvant treatment. Pre-treatment (PRE) samples were obtained on the same day and just before the start of the first cycle of chemotherapy. Post-treatment (POST) samples were obtained in 54 patients after six cycles of chemotherapy (carboplatin plus docetaxel) combined with trastuzumab and pertuzumab, approximately 18 weeks of treatment. These samples included 46 patients from the pCR group and 8 patients from the no-pCR group. No association was found between achieving pCR and clinical or pathological features known for their prognostic value. Neither a Ki67 > 20 (*p* = 0.6313), HR expression (*p* = 0.1887), nor the presence of positive lymph nodes (*p* = 0.3019) was able to explain the probability of response in this population.

### 2.2. Variables in PRE Samples That Were Differentially Expressed between Response Groups

Considering the high response rate obtained in this study, we first evaluated whether there is any association between the treatment response and the basal Complete Blood Counts (CBCs) in the patient cohorts. CBC analysis revealed no differences in total leukocyte counts between the response groups (see Supplementary Table S1).

**Figure 1:**
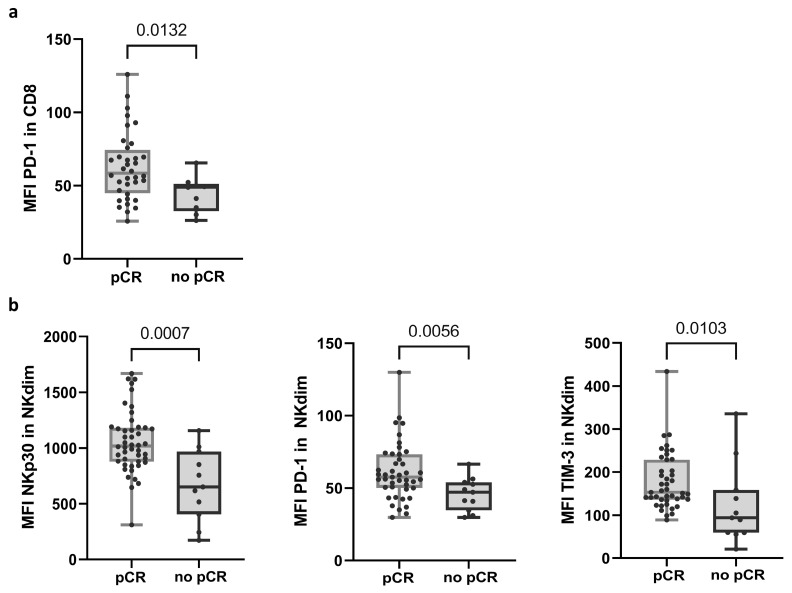
Variables in PRE samples that exhibited differential expression between response groups. (**a**) Expression of PD-1 in CD8 T cells. (**b**) Expression of NKp30, PD-1, and TIM-3 receptors in NKdim cell population. MFI was calculated as the average fluorescence intensity of the antibody–fluorophore complex bound to the molecule of interest in each cell. Each dot represents one sample in the box with the maximum and minimum. Mann–Whitney test was used to determine statistical significance. MFI: mean fluorescence intensity. pCR: pathologic complete response.

Flow cytometry (FC) analysis showed that PD-1 expression, shown as the mean fluorescence intensity (MFI) in CD8 T cells, differentiated response groups (58.45; *n* = 36 in the pCR group and 48.90; *n* = 9 in no-pCR group) (Figure 1a and Supplementary Table S2; see calculation of MFI in Material and Methods). In NK cells, three receptors showed significant differences in MFI between the response groups. Within NK cells, there are two main subpopulations: the less mature and less frequent NK CD56bright (NKbright) cells in PB, and CD56dim (NKdim) cells, which are capable of performing ADCC [30]. The no-pCR group presented a lower expression of NKp30, PD-1, and TIM-3 receptors in the NKdim subset (Figure 1b and Supplementary Table S3). In the NKbright subset, a lower expression of receptor NKp44 was observed.

### 2.3. Variations in Leukocytes and Immune Populations after Anti-HER2 Therapy 

**Figure 2:**
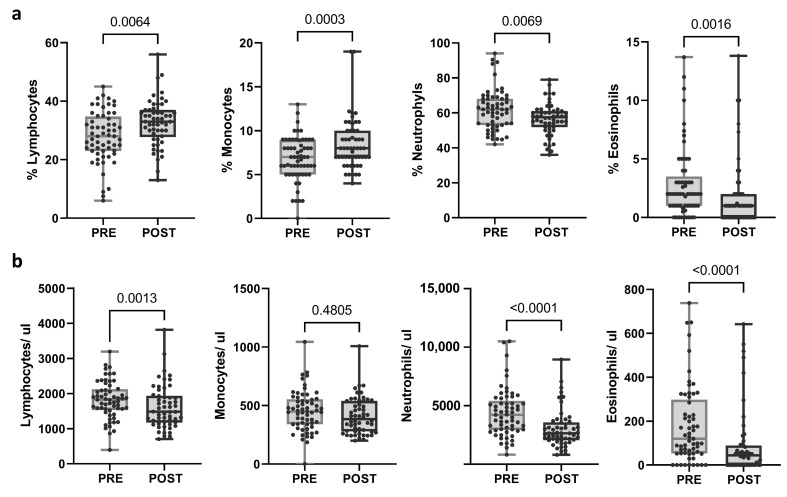
Changes in blood populations (lymphocytes, neutrophils, monocytes, and eosinophils) after treatment. (**a**) Proportions and (**b**) absolute counts calculated as (percentage of population/100) × total leukocytes. Each dot represents one sample in the box with the maximum and minimum. Paired *t*-test (when normal) and Wilcoxon matched-pairs non-parametric signed rank test were used to determine statistical significance.

The same immunological parameters were studied in post-treatment samples and compared with pre-treatment samples. The proportion of lymphocytes and monocytes was significantly augmented (*p* = 0.0064 and *p* = 0.003, respectively), whereas the proportion of neutrophils and eosinophils decreased after the treatment (*p* = 0.0069 and *p* = 0.0016, respectively). Moreover, a significant reduction in the total count of lymphocytes, neutrophils, and eosinophils was observed after neoadjuvant treatment (*p* = 0.013; *p* < 0.0001; *p* < 0.0001, respectively) (Figure 2; Supplementary Table S4). When categorized into response groups, the results were consistent within the pCR group. However, no significant changes were noted in the no-pCR group, except for eosinophil levels (Supplementary Figure S1a).

### 2.4. Changes in T and NK Cell Compartments

Twenty populations and subpopulations of T and NK lymphocytes were analyzed by FC, and several changes were observed after treatment (Supplementary Tables S2 and S5).

Generally, the proportion of T lymphocytes increased (*p* = 0.0002), while the proportion of NK cells decreased (*p* = 0.0071). We found a significant reduction in the proportion of the effector memory (EM) subset within the CD8 T cell population, accompanied by an increase in the proportion of the terminal effector memory (TEM) and HLA-DR subsets. Moreover, after treatment, we detected a significant reduction in the proportion of the EM subset within the CD4 T cell population (Supplementary Table S5). These significant changes were not captured when the data were separated into response groups, except for CD3 T cells, which increased in the responder group (Supplementary Figure S1b).

**Figure 3:**
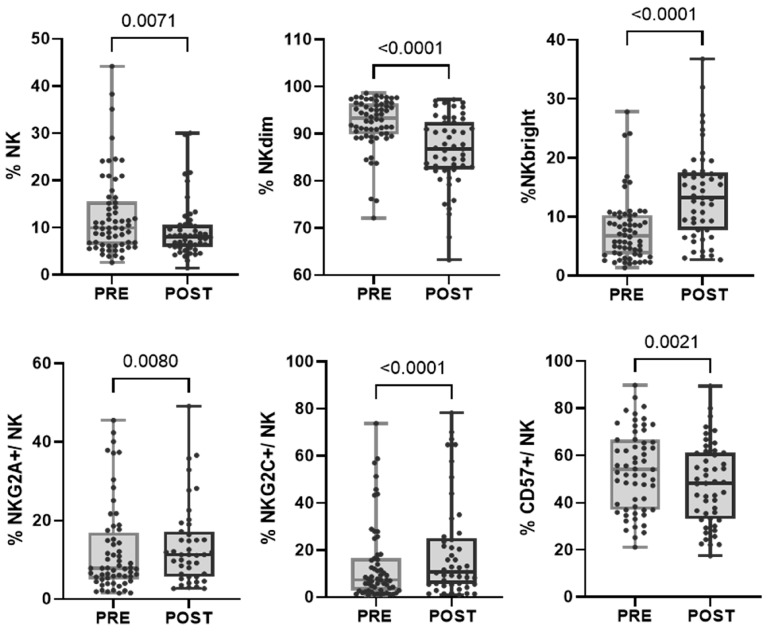
Changes in the proportion of NK cell subpopulations after treatment. Each dot represents one sample in the box with the maximum and minimum. Wilcoxon matched-pairs non-parametric signed rank test was used to determine statistical significance.

In the evaluated cohort of patients, we found that, after treatment, within the NK cell population, there was an increase in the proportion of more immature cells characterized by a higher expression of CD56 (NKbright) and the NKG2A receptor and a decrease in the proportion of mature NKdim and CD57+ cells. However, the subpopulation of adaptive NK cells (CD57+NKG2C+) increased after treatment, as we previously reported (Bordignon 2023) (Figure 3 and Table 2). When divided into response groups, both cohorts showed a persistent increase in NKbright cells and a decrease in NKdim cells (Supplementary Figure S1c).

**Figure 4:**
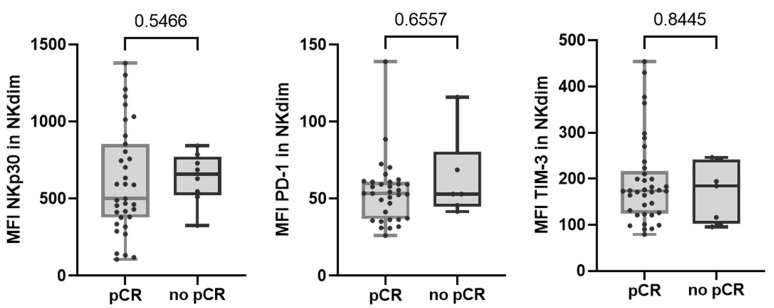
Expression of NKp30, PD-1, and TIM-3 receptors in NKdim cell population post-treatment in both response and non-response groups. MFI was calculated as the average fluorescence intensity of the antibody–fluorophore complex bound to the molecule of interest in each cell. Each dot represents one sample in the box with the maximum and minimum. Mann–Whitney test was used to determine statistical significance. MFI: mean fluorescence intensity.

Regarding altered NK cell receptors in no-pCR group, we observed that none of them were significantly augmented after therapy. We also observed that none of the NK cell receptors that were differentially expressed between response groups maintained their differences after treatment (Figure 4).

### 2.5. Changes in Functional Performance of NK Cells after Treatment

**Figure 5:**
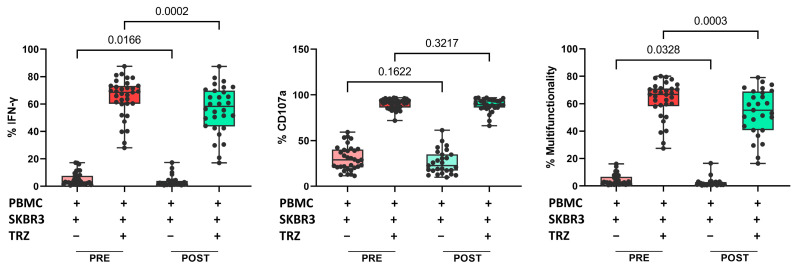
Proportion of IFN-γ production (**left**), proportion of CD107a positive cells (**center**), and multifunctionality (**right**) in cocultures of PBMCs plus SKBR3 cells with or without trastuzumab. Paired *t*-test was used to determine statistical significance. Each dot represents one sample in the box with the maximum and minimum. The results are expressed as the percentage of IFN-γ+ or CD107a+ cells after gating on NKdim cell subset. PRE samples are depicted in red and POST samples in green. Dark colors indicate addition of trastuzumab. PBMCs: peripheral blood mononuclear cells. TRZ: trastuzumab.

Finally, we evaluated whether the treatment affected NK cell functionality. Interferon-gamma (IFN-γ) production and CD107a expression in NK cells were examined in PRE and POST samples from 34 patients. We found a significant reduction in direct IFN-γ production against SKBR3 cells (a human BC cell line that overexpresses *HER2*: HTB-30™ ATCC), as well as in the presence of trastuzumab. However, we did not find significant differences in the percentage of CD107a-positive cells after treatment. The percentage of multifunctionality (production of IFN-γ plus degranulation) was significantly lower after treatment in both conditions, with or without trastuzumab (Figure 5). There were no changes in functionality associated with the response groups.

## 3. Discussion

In the INMUNOHER study, we assessed whether immune cell profiles detected in PB might influence antibody-mediated therapy responses before and after treatment. The purpose of this study was to examine whether peripheral composition reflects immune function and whether it changes differently depending on the response group. We also evaluated the effects of treatment on CBCs and on more than 20 immune subpopulations of NK and T cells from peripheral blood mononuclear cells (PBMCs). The INMUNOHER study included 62 patients with early-stage HER2+ BC. The proportion of patients achieving a pCR was over 82%, resulting in an imbalance between the two groups: 51 patients achieved pCR, while 11 patients did not. This disparity in the response rate observed in our patient cohort may be due to the addition of pertuzumab in neoadjuvant therapy. A recent study showed that the pCR rate was significantly increased in patients receiving pertuzumab with trastuzumab in neoadjuvant chemotherapy (49% vs. 62%), while in HER-2+ subtypes, the response rate was 16% vs. 85% [31].

Clinical and pathological characteristics have been associated with treatment response in numerous studies [16,32,33,34]. However, in our study, neither clinical nor pathological characteristics recognized as prognostic factors were found to be correlated with the response. This lack of association could also be due to the imbalance between the response groups.

Multiparametric FC analysis revealed subtle differences between response groups when we explored innate and adaptive subpopulations. We observed substantial variations in NK cell subsets between the response groups. Patients who achieved pCR showed a higher expression of the natural cytotoxicity receptor NKp30, which plays a key role in the activation of NK cells against target cells and in the crosstalk between NK cells and dendritic cells [35]. In addition, NK cells from patients with pCR expressed higher levels of TIM-3 and PD-1, which was also observed in CD8 T cells. Contrary to what is known for T lymphocytes, the expression of these markers in NK cells is not necessarily linked to exhaustion. Reports from several studies on different tumor models have shown that PD-1+ NK cells exhibit high functional activity, whereas PD-1- NK cells appear rather anergic [36]. Similarly, TIM-3 expression in NK cells has also been associated with strong cytotoxicity and cytokine production [37]. Since these differences have been observed in samples prior to treatment; they could indicate an intrinsically more active immune system, potentially contributing to achieving pCRs.

Among patients with primary BC, Muntasell et al. [38] found that the number of circulating CD57+ NK cells was inversely correlated with pCRs to HER2-specific antibody treatment. Our results differ primarily due to their patients’ treatment and pCR rates. All our patients received trastuzumab and pertuzumab along with chemotherapy, resulting in an 82.3% response, whereas Muntasell’s study had only 31% of patients receiving the same treatment, resulting in a 27% pCR rate.

The large number of patients studied and the homogeneous sample population allowed us to find robust changes after neoadjuvant therapy. The proportions of lymphocytes and monocytes significantly increased, while the proportions of neutrophils and eosinophils decreased; also, the absolute numbers of lymphocytes, neutrophils, and eosinophils were reduced after treatment, as measured by CBC. FC analyses of about 20 subsets of T and NK cells revealed that treatment increased the proportion of CD3 T cells within lymphocytes but did not affect the proportion of cytotoxic or helper lymphocytes. Muraro et al. [39] performed immunophenotyping on PB from HER2+ BC patients who received neoadjuvant chemotherapy combined with trastuzumab and did not observe the increase in the percentage of T lymphocytes after therapy that we saw in our cohort. However, our cohort showed an increase in the proportion of TEM and HLA-DR CD8 T cells, indicating the greater activity of these populations during therapy compared with Muraro’s findings, where naive CD8 T cells increased post-treatment. Similar results were obtained for CD4 T cells, with a decrease in EM populations. After treatment, the proportion of NK cells decreased, and their phenotype became more immature. We observed a reduction in CD57+ NK cells alongside an increase in NKbright and NKG2A+ NK cells. Notably, adaptive or memory NKG2C+ NK cells increased, consistent with our previous results [40]. Gaynor et al. [41], in the phase II neoadjuvant clinical trial ICORG10-05 (NCT01485926), compared immune cell profiles in HER2+ BC patients treated with chemotherapy with trastuzumab, lapatinib, or both. They did not find significant differences in the immune cell populations from pre- or post-treatment samples within the pCR cohort. In contrast, the no-pCR cohort showed an increase in CD3, CD4, and CD8 T cells and decreased CD56 NK cells, CD14+ monocytes, and CD19+ B cells after treatment. This study showed an imbalance between response groups in the opposite direction to our findings. The pCR group in that study was nearly three times smaller than the no-pCR group, likely contributing to the significant differences observed between the PRE and POST samples, being more evident in the larger no-pCR group. Similarly, we found that differences were predominantly observed in the pCR group, which, in our case, was the larger group. However, changes in the no-pCR cohort followed the same trend as in the pCR cohort, possibly because they actually presented partial responses. In this regard, we hypothesize that those with a partial response would be immunologically similar to patients with a complete response.

Evaluation of the NK cells’ ex vivo functionality showed a reduction in IFN-γ production capacity after treatment, both when PBMCs were co-cultured with SKBR3 HER2+ cells alone or in the presence of trastuzumab. This reduction could be related to the more immature phenotype of the NK cells after treatment. We did not detect changes in the percentage of degranulation. However, the combination of these variables showed a reduction in multifunctionality, whether in the presence or absence of trastuzumab, after treatment. The Gaynor study reported no significant alteration to direct cytotoxicity against SKBR3 cells post-treatment but observed a significant decrease in trastuzumab-induced ADCC mediated by PBMCs [41].

Understanding immune cell phenotype and activation status (cellular immunome) in tumors and blood is crucial for assessing patient responses and developing biomarkers of therapy response. Our study was limited by the small sample size of patients who did not achieve pCR, which may have affected the evaluation of peripheral immune cell subsets and led to an unbalanced distribution of immune subset positivity in each arm. Nonetheless, we observed interesting results regarding NK cell activation that could differentiate response groups. The observations in the PRE samples were descriptive rather than predictive. Both CD8 T cells and NK cells showed a higher PD-1 expression in the pCR group, which could indicate active populations within effector compartments [36,42]. However, these findings are preliminary and require confirmation in future studies due to the limited statistical power. Further conclusions could be drawn from our study regarding changes generated by therapy in the peripheral immunological context of patients.

## 4. Materials and Methods

### 4.1. Patients Samples

Patients from three institutions in Buenos Aires, Argentina (Alexander Fleming Institute, CEMIC University Hospital, and Austral University Hospital), were invited to participate in the study. All patients who agreed to participate in the INMUNOHER study signed an informed consent. Heparinized PB samples at baseline were obtained on the same day and just before the start of the first cycle of chemotherapy administration (PRE sample), with an 8 mg/kg trastuzumab loading dose and 6 mg/kg every 21 days, an 840 mg pertuzumab loading dose and 420 mg every 21 days, carboplatin AUC5 and docetaxel at 80 mg/m^2^ every 21 days (all of them for 6 cycles). The POST sample was obtained after completion of treatment, prior to surgery. Sixty-two patients were administered granulocyte-colony-stimulating factor at the last cycle of prior chemotherapy. Samples were obtained between May 2018 and May 2022. The responder group was considered the pCR group, defined by patients who showed a complete response, i.e., no remaining invasive tumor cells at the time of surgery in the breast and the axilla, independently of in situ residual disease. The non-responder group was defined by patients who showed remaining tumor cells after surgery, without pCR. Patients who received less than 6 cycles of treatment were not considered for response assessment.

### 4.2. CBC and FC Analysis on PB

Neutrophils, lymphocytes, monocytes, and eosinophils were evaluated in PB samples using an automated cell blood counter. The absolute count was calculated as follows: (percentage of population/100) × total leukocytes. PBMCs were isolated through a Ficoll–Paque density gradient (GE Healthcare, Chicago, IL, USA) and processed on the same day for phenotyping or frozen for subsequent functional experiments. For T and NK cell phenotyping, 2.5 × 10^5^ fresh PBMCs were washed with staining buffer (PBS with 2% FBS), incubated with the appropriate mAbs (Supplementary Table S6) for 30 min at 4 °C, and then washed twice with staining buffer. Isotype-matched irrelevant mAbs were used as negative controls. Lymphocytes were gated using forward scatter-area (FSC-A) vs. side scatter-area (SSC-A) plot, and single cells using FSC-A vs. forward scatter-height (FSC-H). Lymphocyte subsets were defined as CD3−CD56+ NK cells, CD4+CD3+ helper T cells, and CD8+CD3+ cytotoxic T cells. Data acquisition was performed using a FACSCanto II cytometer and the FACSDiva v8.0.1 software (BD Biosciences, San Jose, CA, USA). Data analysis was performed with the FlowJo v10.6.2 software (Ashland, OR, USA). A representative gating strategy is shown in Supplementary Figure S2. The MFI was calculated as the average fluorescence intensity of the antibody–fluorophore complex bound to the molecule of interest in each cell.

### 4.3. CD107a Degranulation and IFN-γ Production Assay

For 22 patients with pCR and 10 without pCR, measurements of NK cell degranulation and intracellular IFN-γ were analyzed in baseline PBMC samples after 6 h coculture with trastuzumab-coated SKBR3 cells (ATCC, Manassas, VA, USA) using standard FC protocols. Briefly, PBMCs were thawed and allowed to recover for one hour with DNase treatment. Subsequently, they were incubated overnight in complete RPMI medium supplemented with 0.3 ng/mL of IL-15. Following this, 2.5 × 10^5^ PBMCs were cultured with SKBR3 cells in the presence of 10 µg/mL of trastuzumab or human IgG1 isotype control mAb in 96-well plates with RPMI medium (Thermo Fisher, Waltham, MA, USA). The number of target cells was calculated according to the percentage of NK cells, so the co-cultures were performed at a 1:1 NK cell:BC cell (NK:BC) ratio. Cells were incubated for 6 h at 37 °C in 5% CO_2_, with the addition of PECy7 anti-CD107a (clone H4A3, BD) from the beginning of the assay and Protein Transport Inhibitor (Golgi Stop, BD) after the first hour. Cells were harvested, washed, and labeled with Fixable Viability Stain 510 (BD) for 15 min at room temperature, washed with staining buffer, and labeled with BV421 anti-CD56 and APC-H7 anti-CD3 (clones NCAM16.2 and SK7, BD) for 15 min at room temperature. After that, cells were fixed and permeabilized using a fixation and permeabilization kit (BD) according to the manufacturer’s protocol and then labeled with PE anti-IFN-γ (clones 4S.B3, BD) for 30 min at 4 °C. Cells were acquired in a FACSCanto II flow cytometer and the FACSDiva v8.0.1 software (BD), and the data were analyzed using the FlowJo v10.6.2 software. A representative gating strategy is shown in Supplementary Figure S3. The results are expressed as the percentage of IFN-γ+ or CD107a+ cells after gating in NKdim cell subsets. Basal degranulation and IFN-γ production were determined in the absence of target cells and are shown in the corresponding graphs.

### 4.4. Statistical Analysis

GraphPad Prism 9.0 (San Diego, CA, USA) was used for graphs and paired *t*-test analysis. Comparisons between samples were performed using Mann–Whitney tests and nonparametric one-way ANOVA with Dunns and Kruskal–Wallis post-tests for multiple comparison analysis. To compare variables between paired groups, when the normal distribution was met, a paired *t*-test was performed. Otherwise, the Wilcoxon test was performed. In all figures, reported *p*-values are two-tailed, and *p* < 0.05 was considered significant. The independence of clinical or pathological features was tested with the χ2 test.

## Figures and Tables

**Table 1:** Clinical characteristics of patients.

Characteristics	*n* (%)	
	pCR	no-pCR
*n*	51	11
Age: median (IQR)	50 (41–57)	58 (44–64)
Histological type		
NST	48 (94.1)	10 (90.9)
Lobular	1 (1.2)	1 (9.1)
Others	2 (3.9)	0 (0)
Histological grade		
1	0 (0)	1 (9.1)
2	22 (43.1)	4 (36.4)
3	21 (41.2)	4 (36.4)
ND	8 (15.7)	2 (18.2)
Stage		
1	5 (9.8)	2 (18.2)
2	33 (64.7)	7 (63.6)
3	13 (25.5)	2 (18.2)
HR+/HER2+	26 (51)	8 (72.7)
HR−/HER2+	25 (49)	3 (27.3)
Ki67: median (IQR)	40 (24–45)	40 (25–50)
Ki67 > 20%	38 (74.5)	10 (90.9)

IQR: interquartile range. ND: non-determined. NST: no special type.

**Table 2:** NK cell populations after treatment. Wilcoxon matched-pairs non-parametric signed rank test was used to determine statistical significance.

	PRE	POST	
	*n*	*n*	*p*-Value
% CD16 (NK)	59	47	0.0642
MFI CD16 (NK)	41	30	0.0815
**% CD16 (NKdim)**	**59**	**47**	**0.0436**
MFI CD16 (NKdim)	41	30	0.1936
**% CD57 (NK)**	**59**	**49**	**0.0021**
**MFI CD57 (NK)**	**55**	**45**	**0.0026**
**% CD57 (NKdim)**	**59**	**49**	**0.0439**
**MFI CD57 (NKdim)**	**55**	**45**	**0.0358**
% NKp30 (NK)	60	47	0.6089
MFI NKp30 (NK)	57	43	0.5024
% NKp30 (NKdim)	60	47	0.5842
MFI NKp30 (NKdim)	57	43	0.0968
%NKp44 (NK)	57	47	0.2495
**MFI NKp44 (NK)**	**55**	**43**	**0.0046**
**%NKp44 (NKdim)**	**57**	**47**	**0.0197**
**MFI NKp44 (NKdim)**	**55**	**43**	**0.0010**
**%NKG2C (NK)**	**43**	**39**	**<0.0001**
**MFI NKG2C (NK)**	**39**	**38**	**0.0111**
**%NKG2C (NKdim)**	**43**	**39**	**<0.0001**
**MFI NKG2C (NKdim)**	**39**	**38**	**0.0097**
% CD25 (NK)	61	47	0.8967
**MFI CD25 (NK)**	**58**	**43**	**0.0160**
% CD25 (NKdim)	61	47	0.7028
**MFI CD25 (NKdim)**	**58**	**43**	**0.0001**
**% NKG2A (NK)**	**60**	**43**	**0.0080**
MFI NKG2A (NK)	57	39	0.1635
% NKG2A (NKdim)	60	43	0.0581
**MFI NKG2A (NKdim)**	**57**	**39**	**0.0068**
% TIM-3 (NK)	59	48	0.6273
MFI TIM-3 (NK)	54	45	0.6866
% TIM-3 (NKdim)	59	48	0.6129
MFI TIM-3 (NKdim)	54	45	0.8600
% PD-L1 (NK)	62	50	0.7467
**MFI PD-L1 (NK)**	**59**	**46**	**0.0026**
% PD-L1 (NKdim)	62	50	0.6135
**MFI PD-L1 (NKdim)**	**59**	**46**	**0.0086**
% PD-1 (NK)	55	41	0.6374
**MFI PD-1 (NK)**	**54**	**39**	**0.0042**
% PD-1 (NKdim)	54	41	0.9629
**MFI PD-1 (NKdim)**	**54**	**39**	**0.0040**

MFI: mean fluorescence intensity. Populations that presented significant differences are indicated in bold.

## Data Availability

The datasets generated and/or analyzed during the current study are available from the corresponding author upon reasonable request.

## Supplementary Materials

The following supporting information can be downloaded at https://www.mdpi.com/article/10.3390/ijms25179268/s1.
