# Supplementary material for: In-Depth Analysis of the Peripheral Immune Profile of HER2+ Breast Cancer Patients on Neoadjuvant Treatment with Chemotherapy Plus Trastuzumab Plus Pertuzumab
Source: Int J Mol Sci. 2024 Aug 27;25(17):9268. doi: 10.3390/ijms25179268 (PMC11395157; doi:10.3390/ijms25179268)
Supplement: Supplementary file 1 [file ijms-25-09268-s001.zip › Supplementary Tables.pdf]

Supplementary Table S1. CBC in response groups. Unpaired t-test and Mann-Whitney test were used to determine statistical significance.

	pCR	no pCR		
	**(mean ± SD; n)**	**(mean ± SD; n)**	**p value**	**Statistic test**
% Lymphocytes	27.98 ± 8.917; 49	27.36 ± 8.152; 11	0.8344	unpaired t test
Lymphocytes/µl	1809.95 ± 556.55; 49	1844.27 ± 451.39; 11	0.8495	unpaired t test
% Monocytes	6.714 ± 2.442; 50	7.282 ± 2.862; 11	0.5011	unpaired t test
Monocytes/µl	445.9 ± 162.5; 49	497.3 ± 225.8; 11	0.3824	unpaired t test
	**(median, n)**	**(median, n)**		
% Eosinophils	2; 50	2; 11	0.8716	Mann-Whitney test
Eosinophils/µl	127.8; 49	120; 11	0.8532	Mann-Whitney test
% Basophils	0.2; 50	0.1; 11	0.9650	Mann-Whitney test
Basophils/µl	26.50; 46	12; 11	0.8830	Mann-Whitney test
% Neutrophils	62; 49	62; 11	0.9512	Mann-Whitney test
Neutrophils/µl	4225; 49	4246 ± 2246; 11	0.8770	Mann-Whitney test

Supplementary Table S2. CD4 and CD8 T lymphocyte populations in response groups. Mann-Whitney test was used to determine statistical significance.

	pCR	no pCR	
	**(median, n)**	**(median, n)**	**p value**
% CD4	71.90; 48	70.30; 11	0.9580
% TIM-3 (CD4)	7.8; 48	9.160; 11	0.6402
MFI TIM-3 (CD4)	42.40; 43	42.50; 11	0.9789
% PD-1 (CD4)	9.900; 42	8.160;11	0.7744
MFI PD-1 (CD4)	56.20; 41	47.70; 11	0.0794
% HLA-DR (CD4)	8.345; 43	8.31; 11	0.5102
MFI HLA-DR (CD4)	36.40; 43	36.70; 11	0.9283
%CD28 (CD4)	98.70; 47	98.60; 11	0.3076
MFI CD28 (CD4)	2293; 42	2419; 11	0.9780
% CD8	19.36; 48	18.56; 11	0.8485
% TIM-3 (CD8)	13.20; 48	11; 11	0.7193
MFI TIM-3 (CD8)	42.9; 43	47.1; 11	0.9366
% PD-1 (CD8)	9.82; 37	11.30; 9	0.8668
**MFI PD-1 (CD8)**	**58.45; 36**	**48.90; 9**	**0.0132**
% HLA-DR (CD8)	19.00; 48	19.50; 11	0.6688
MFI HLA-DR (CD8)	48.60; 43	47.00; 11	0.7642
%CD28 (CD8)	75.40; 47	75.40; 11	0.6351
MFI CD28 (CD8)	1020; 42	1164; 11	0.7375

Supplementary Table S3. NK cells subpopulations in response groups. Mann-Whitney test was used to determine statistical significance.

	**pCR (median, n)**	**no pCR (median, n)**	**p value**
% CD16 (NK)	93.10; 47	92.30; 11	0.8641
MFI CD16 (NK)	10151; 31	12824; 9	0.3749
% CD16 (NKdim)	97.50; 47	97.20;11	0.9415
MFI CD16 (NKdim)	10426; 31	14002; 9	0.3709
% CD57 (NK)	51.80; 47	55.60; 11	0.9402
MFI CD57 (NK)	277; 43	516; 11	0.8461
% CD57 (NKdim)	58.80; 47	62.90; 11	0.9184
MFI CD57 (NKdim)	413; 43	594; 11	0.8158
% NKp30 (NK)	92.20; 49	94.50; 11	0.3566
MFI NKp30 (NK)	591; 46	713; 11	0.7009
% NKp30 (NKdim)	91.60; 49	94.80; 11	0.2658
**MFI NKp30 (NKdim)**	**1019; 46**	**651; 11**	**0.0007**
%NKp44 (NK)	4.940; 47	4.395; 10	0.4372
MFI NKp44 (NK)	38.30; 45	37.85; 10	0.2029
%NKp44 (NKdim)	4.300; 47	3.955; 10	0.4527
MFI NKp44 (NKdim)	38.30; 45	38.20; 10	0.2679
%NKG2C (NK)	7.600; 47	5.780; 11	0.6527
MFI NKG2C (NK)	24; 42	22.40; 11	0.3884
%NKG2C (NKdim)	6.740; 47	5.380; 11	0.8887
MFI NKG2C (NKdim)	24.70; 42	22.00; 11	0.3591
% CD25 (NK)	5.59; 50	5.50; 11	0.9225
MFI CD25 (NK)	20.30; 47	19.10; 11	0.1781
% CD25 (NKdim)	4.525; 50	4.370; 11	0.9484
MFI CD25 (NKdim)	18.30; 47	16.60; 11	0.1479
% NKG2A (NK)	7.84; 49	7.63; 11	0.8928
MFI NKG2A (NK)	33.85; 46	30.70; 11	0.1344
% NKG2A (NKdim)	7.52; 49	7.63; 11	0.6240
MFI NKG2A (NKdim)	33.50; 46	30.70; 11	0.1373
% TIM-3 (NK)	62.10;48	48.60; 11	0.7190
MFI TIM-3 (NK)	154; 43	169; 11	0.8781
% TIM-3 (NKdim)	64.65; 48	49.90; 11	0.5177
**MFI TIM-3 (NKdim)**	**153; 43**	**94.14;11**	**0.0103**
% PD-L1 (NK)	4.0705; 50	5.370; 11	0.5201
MFI PD-L1 (NK)	25.60; 47	19.90;11	0.1370
% PD-L1 (NKdim)	5.15;50	5.68; 11	0.2508
MFI PD-L1 (NKdim)	27.40; 47	22.80; 11	0.1749
% PD-1 (NK)	1.47; 44	1.16;11	0.5161
**MFI PD-1 (NK)**	**56.40; 43**	**47.20; 11**	**0.0044**
% PD-1 (NKdim)	1.60;43	1.260;11	0.5998
**MFI PD-1 (NKdim)**	**57.60; 43**	**47.20; 11**	**0.0056**

Supplementary Table S4. Changes in CBC after treatment. Paired t-tests and Wilcoxon matched-pairs signed rank test were used to determine statistical significance.

	PRE	POST		
	(mean ± SD; n)	(mean ± SD; n)	p value	Statistic test
**%Lymphocytes**	**27.87 ± 8.71; 60**	**32.70 ± 7.92; 54**	**0.0064**	**Paired t test**
	(median; n)	(median, n)		
**%Neutrophyls**	**62.00; 60**	**57.50; 54**	**0.0069**	**Wilcoxon test**
**%Monocytes**	**7.000, 61**	**8.000; 54**	**0.0003**	**Wilcoxon test**
**%Eosinophils**	**2.000; 61**	**1.000; 54**	**0.0016**	**Wilcoxon test**
%Basophils	0.200; 61	1.000; 54	0.1907	Wilcoxon test

Supplementary Table S5. Changes in T lymphocytes after treatment. Wilcoxon matched-pairs signed rank test was used to determine statistical significance.

	PRE	POST	
	(median; n)	(median; n)	p value
**%CD3**	**72.00; 59**	**78.90; 50**	**0.0002**
%CD4	**71.50; 59**	75.55; 50	0.5325
%TIM-3 (CD4)	7.990; 59	7.530; 49	0.6129
MFI TIM-3 (CD4)	42.45; 54	44.10; 43	0.6839
%PD-1 (CD4)	9.810; 53	10.60; 42	0.4505
MFI PD-1 (CD4)	53.05; 52	49.80; 40	0.1083
%HLA-DR (CD4)	8.330; 59	7.750; 49	0.0740
**MFI HLA-DR (CD4)**	**36.45; 54**	**33.90; 45**	**0.0004**
%CD28 (CD4)	98.70; 58	98.10; 49	0.2143
MFI CD28 (CD4)	2350; 53	2269; 45	0.9040
%NAIVE (CD4)	39.80; 58	44.55; 50	0.2878
%CM (CD4)	25.55; 58	30.85; 50	0.4406
**%EM (CD4)**	**24.25; 58**	**17.35; 50**	**0.0220**
%TEM (CD4)	4.835; 58	4.430; 50	0.9211
%CD8	16.70; 59	14.20; 50	0.5164
%TIM-3 (CD8)	12.20; 59	12.80; 49	0.3292
MFI TIM-3 (CD8)	46.25; 54	48.30; 43	0.2430
% PD-1 (CD8)	9.835; 46	8.850; 37	0.3974
MFI PD-1 (CD8)	55.00; 45	53.70; 35	0.6554
**%HLA-DR (CD8)**	**19.50; 59**	**23.90; 49**	**<0.0001**
**MFI HLA-DR (CD8)**	**47.80; 54**	**48.30; 45**	**0.0057**
%CD28 (CD8)	75.40, 58	70.00, 49	0.1318
MFI CD28 (CD8)	1037; 53	943.0; 45	0.1839
%NAIVE (CD8)	31.35; 58	35.35; 50	0.6345
%CM (CD8)	4.905; 58	5.230; 50	0.9473
**%EM (CD8)**	**21.50; 58**	**17.50; 50**	**0.0113**
**%TEM (CD8)**	**33.30; 58**	**36.15; 50**	**0.0191**

Supplementary Table S6. Ac used for flow cytometry studies.

Target molecule	Fluorochrome	Clon	Company
IgG1	BB515	X40	BD
IgG1	AF647	MOPC-21	BD
IgG1	BV421	X40	BD
IgG2a	FITC	G155-178	BD
IgG1	PE	MOPC-21	BD
IgG1	APC	X40	BD
IgG2a	BV421	G155-178	BD
IgG1	PECy7	MOPC-21	BD
IgG2a	PE	X39	BD
IgG2b	PECy7	MPC-11	Biolegend
CD3	APC-H7	SK7	BD
CD4	PerCPCy5.5	RPA-T4	BD
CD8	PECy7	RPA-T8	BD
CD56	BV421	NCAM 16.2	BD
CD57	FITC	NK-1	BD
TIM-3	BB515	7D3	BD
LAG-3	AF647	T47-530	BD
PD-1	BV421	MIH4	BD
HLA-DR	FITC	G46-6	BD
CD137	PE	4B4-1	BD
CD28	APC	CD28.2	BD
CTLA-4	BV421	BNI3	BD
CCR7	PE	150503	BD
CD45RO	APC	2D1	BD
NKp44	BB515	p488-8	BD
CD25	PECy7	M-A251	BD
NKG2A	PE	# 131411	R&D
NKp30	AF647	P30-15	BD
NKG2C	AF647	# 134591	R&D
PDL-1	PECy7	29E.2A3	Biolegend
CD20	FITC	2H7	BD
HLA-A2	PE	BB7.2	BD
CD16	PE	3G8	BD
CD57	FITC	NK-1	BD
