# Supplementary figures and images for: In-Depth Analysis of the Peripheral Immune Profile of HER2+ Breast Cancer Patients on Neoadjuvant Treatment with Chemotherapy Plus Trastuzumab Plus Pertuzumab
Source: Int J Mol Sci. 2024 Aug 27;25(17):9268. doi: 10.3390/ijms25179268 (PMC11395157; doi:10.3390/ijms25179268)

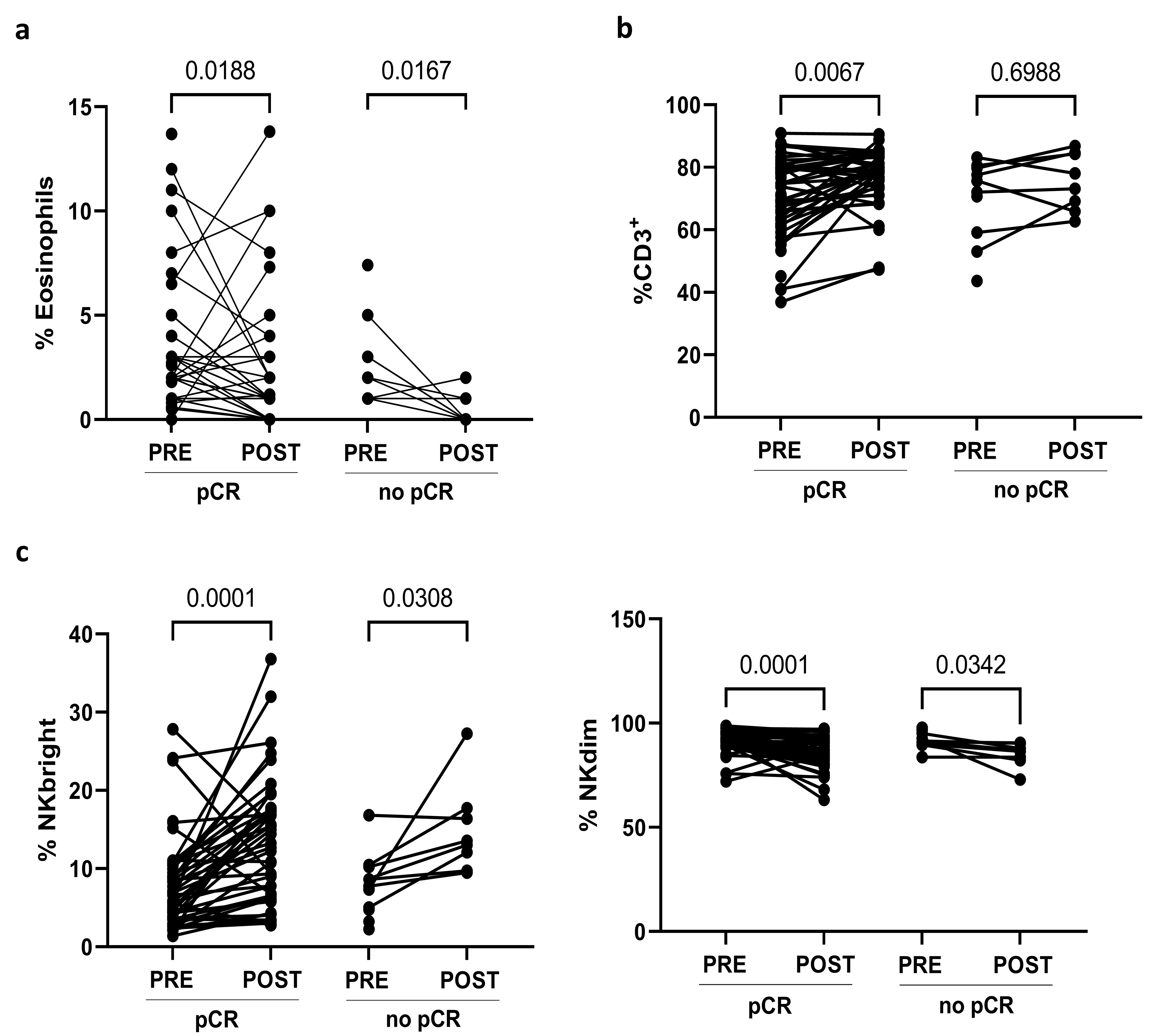

Supplement: Supplementary file 1 [file ijms-25-09268-s001.zip › Supplementary Figure S1. Changes in immune populations after treatment in response groups.tif]

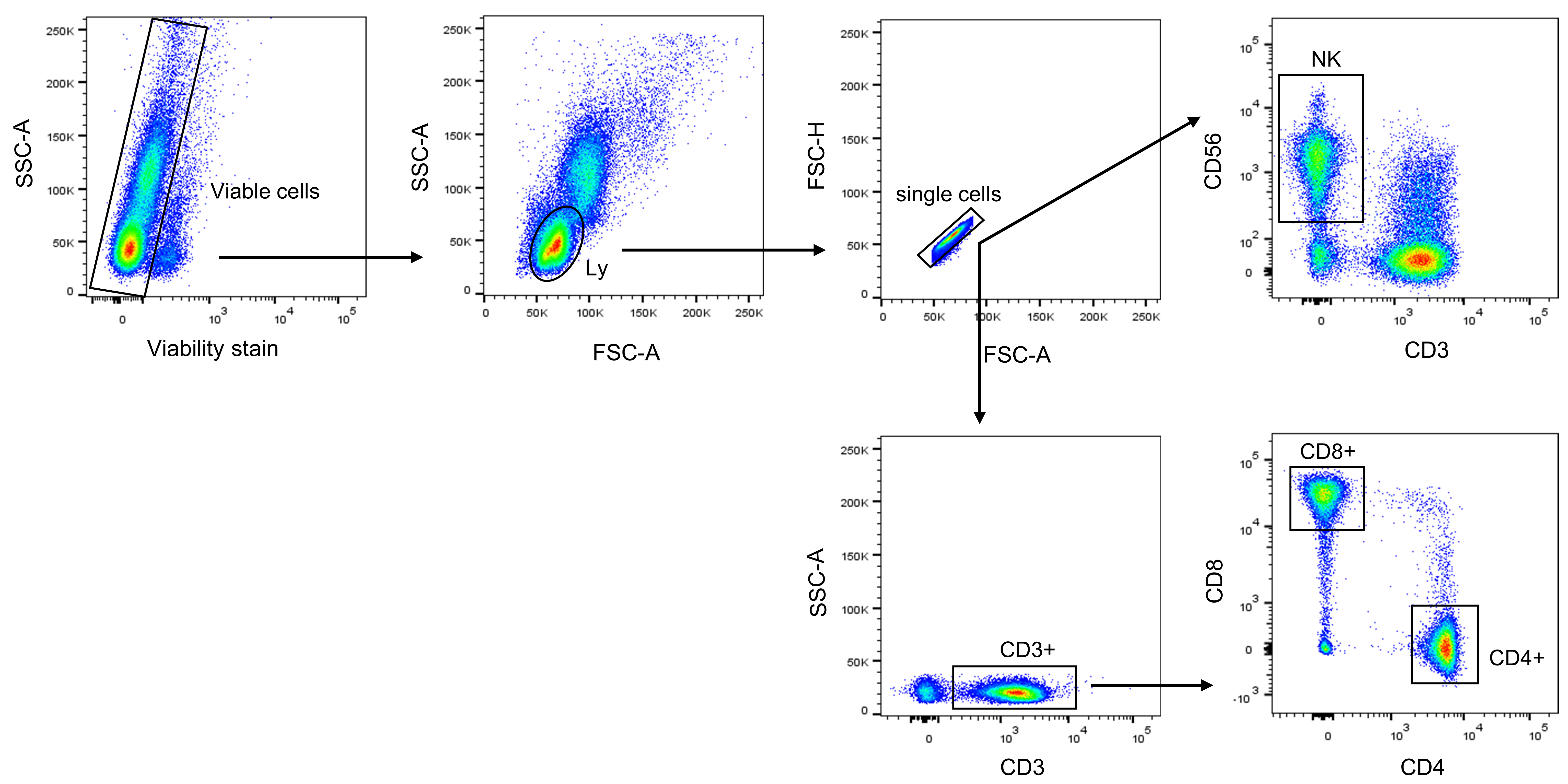

Supplement: Supplementary file 1 [file ijms-25-09268-s001.zip › Supplementary Figure S2. Representative gating strategy.tif]

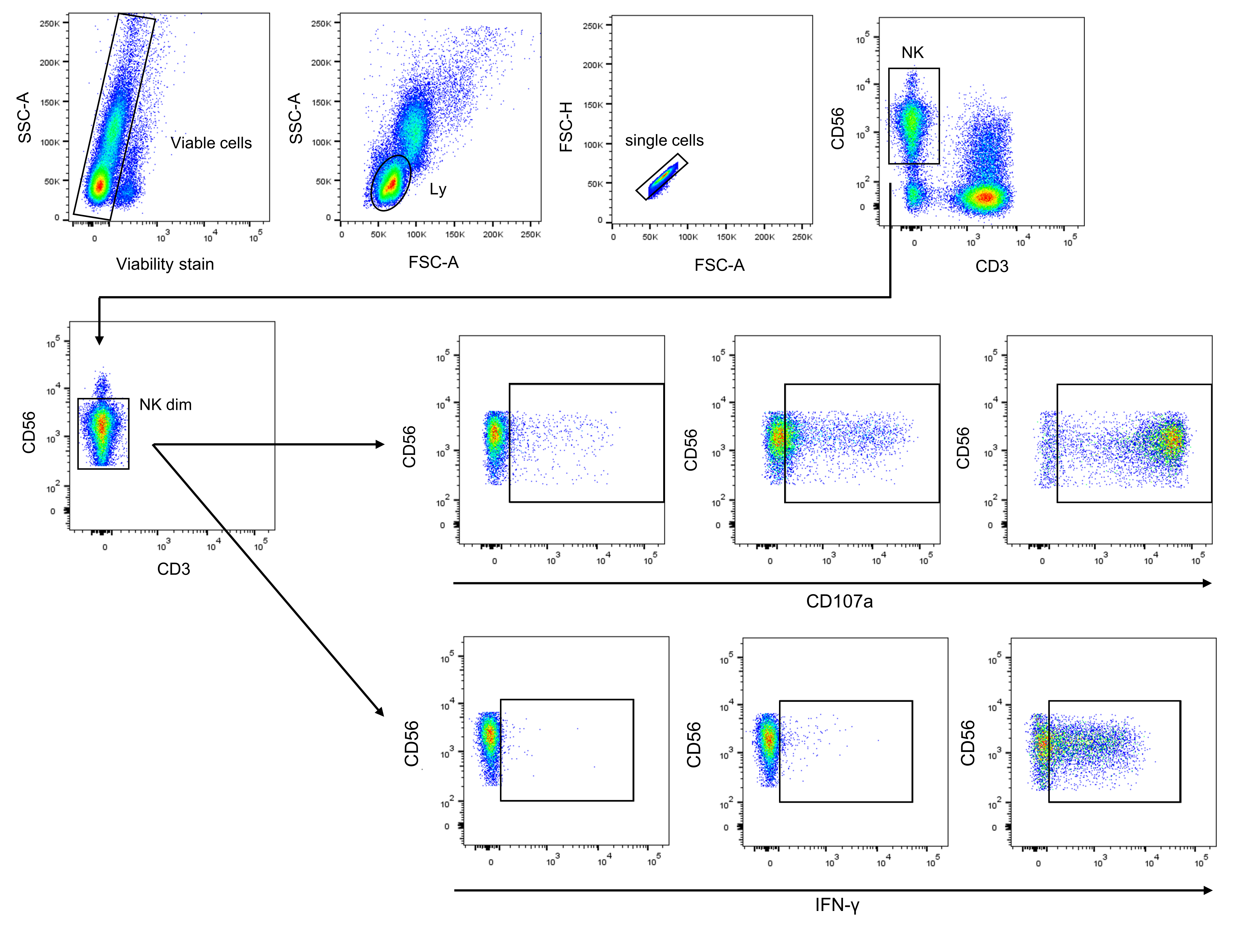

Supplement: Supplementary file 1 [file ijms-25-09268-s001.zip › Supplementary Figure S3. Representative dot plot of CD107a expression and IFN-a├ production.tif]
